# *KAZN* as a diagnostic marker in ovarian cancer: a comprehensive analysis based on microarray, mRNA-sequencing, and methylation data

**DOI:** 10.1186/s12885-022-09747-2

**Published:** 2022-06-16

**Authors:** Songling Zhu, Hongxia Bao, Meng-Chun Zhang, Huidi Liu, Yao Wang, Caiji Lin, Xingjuan Zhao, Shu-Lin Liu

**Affiliations:** 1 Genomics Research Center (State-Province Key Laboratories of Biomedicine-Pharmaceutics of China), College of Pharmacy, Harbin Medical University, Harbin, China; 2 HMU-UCCSM Centre for Infection and Genomics, Harbin Medical University, Harbin, China; 3 Translational Medicine Research and Cooperation Center of Northern China, Heilongjiang Academy of Medical Sciences, Harbin, China; 4 Physical Examination Center, The Second Affiliated Hospital of Harbin Medical University, Harbin, China; 5 Department of Microbiology, Immunology and Infectious Diseases, University of Calgary, Calgary, Canada

**Keywords:** *KAZN*, Ovarian cancer, Methylation, Bioinformatics, Survival

## Abstract

**Background:**

Ovarian cancer (OC) is among the deadliest malignancies in women and the lack of appropriate markers for early diagnosis leads to poor prognosis in most cases. Previous studies have shown that *KAZN* is involved in multiple biological processes during development, such as cell proliferation, differentiation, and apoptosis, so defects or aberrant expression of *KAZN* might cause queer cell behaviors such as malignancy. Here we evaluated the *KAZN* expression and methylation levels for possible use as an early diagnosis marker for OC.

**Methods:**

We used data from Gene Expression Omnibus (GEO) microarrays, The Cancer Genome Atlas (TCGA), and Clinical Proteomic Tumor Analysis Consortium (CPTAC) to investigate the correlations between *KAZN* expression and clinical characteristics of OC by comparing methylation levels of normal and OC samples. The relationships among differentially methylated sites in the *KAZN* gene, corresponding *KAZN* mRNA expression levels and prognosis were analyzed.

**Results:**

*KAZN* was up-regulated in ovarian epithelial tumors and the expression of *KAZN* was correlated with the patients’ survival time. *KAZN* CpG site cg17657618 was positively correlated with the expression of mRNA and the methylation levels were significantly differential between the group of stage “I and II” and the group of stage “III and IV”. This study also presents a new method to classify tumor and normal tissue in OC using DNA methylation pattern in the *KAZN* gene body region.

**Conclusions:**

*KAZN* was involved in ovarian cancer pathogenesis. Our results demonstrate a new direction for ovarian cancer research and provide a potential diagnostic biomarker as well as a novel therapeutic target for clinical application.

**Supplementary Information:**

The online version contains supplementary material available at 10.1186/s12885-022-09747-2.

## Background

Ovarian cancer is one of the most lethal gynecological malignancies in women. Due to the absence of symptoms at early stages and late detection, ovarian cancer is usually fatal. Data from SEER 182010–2016 show that 5-years relative survival for all patients with ovarian cancer is 46.8%, but 5-years relative survival in patients at early-stages is significantly better than in patients at advanced-stages (92.6% vs30.2%) [1]. The most common histological types are epithelial tumors, including high-grade serous carcinoma (HGSOC, accounting for ~ 60%), endometrioid, clear cell, mucinous, and low-grade serous carcinomas [2]. Recent studies report that the different histological subtypes of ovarian cancer may have distinct origins. Defects in *BRCA1* and *BRCA2* are well known genetic risks [3, 4], and mutations in many other genes are also associated with ovarian cancer, such as *MSH2*, *MSH6,* and *TP53* [5–9], but in many cases the involved genetic factors remain unknown. Because of the genetic diversity and histological heterogeneity of ovarian cancer, the application of these studies for clinical diagnosis and effective therapy is rare and further investigation is urgently required [10].

Epigenetic changes are integral to all aspects of cancer genomics, such as DNA methylation, which is among the common epigenetic mechanisms involved in the formation and development of cancer [11, 12]. A large body of studies has shown that aberrant methylation of global DNA or specific genes may affect the progression [13] and prognosis of ovarian cancer [14], but the mechanisms for the aberrant DNA methylations to be involved in ovarian cancer remain largely unknown.

*KAZN* is an evolutionarily conserved gene initially identified in keratinocytes and then found to be widely expressed across different human tissues [15]. A study with mouse eggs demonstrates that the cellular localization of KAZN changes dynamically during the development [16]. KAZN is partially co-localized with desmoplakin and periplakin at desmosome and involved in the interplay between adherens junctions and desmosomes [15]. By binding to actin and intermediate filament, KAZN can affect the cell shape and remodeling of cytoskeletal networks [17]. In the nucleus, KAZN was found to be associated with the cell cycle, gene regulation, and matrix stability. KAZN interacts with apoptotic regulators, ARC and Bax, and plays an important role in apoptosis and cell growth [18]. *KAZN* is up-regulated during keratinocyte terminal differentiation [17] and is dispensable for murine epidermal morphogenesis and homeostasis [19]. Since KAZN is involved virtually in all aspects of cell development, its roles in carcinogenesis can be speculated [20, 21]. Therefore, according to the dynamic changes of *KAZN* expression during cell development and the regulation of gene expression by epigenetics in time and space [22, 23], we postulate that the expression and methylation of *KAZN* are crucial in the occurrence and development of ovarian cancer.

In this study, we conducted a comprehensive analysis on *KAZN*, using the data from GEO, TCGA, GTEx, and CPTAC datasets. We compared *KAZN* expression at levels of mRNA, protein, and DNA methylation, and detected the correlation between *KAZN* expression and survival time of the patients. Our results indicated that the expression and methylation patterns of *KAZN* were closely associated with the oncogenesis of ovarian cancer. These results can contribute to understanding the molecular mechanisms of tumor occurrence and progression and can be used to develop new diagnostic as well as treatment strategies.

## Materials and methods

### Gene expression omnibus (GEO) database

The GEO database (https://www.ncbi.nlm.nih.gov/geo/) is a public storage repository of microarray, methylation, and next-generation sequencing data. We searched the GEO datasets with the keywords “ovarian cancer” and “mRNA” and then filtered the documents by the following criteria: 1, organisms from the *Homo* species; and 2, datasets that contain both the tumor and non-tumor tissues (normal, benign, or para-carcinomas tissue). R package ‘GEOquery’ was used for data download and preprocessing. R package ‘Limma’ was used to normalize and analyze the data. A t-test is a statistical test used to compare the means of two groups. R package ‘ggpubr’ was used to plot the boxplot.

### Comprehensive meta-analysis

R package ‘meta’ was used for performing a comprehensive meta-analysis of GEO data. The analysis of *KAZN* expression in the normal and tumor group was displayed on forest plots that illustrate the standardized mean difference (SMD) and the 95% confidential interval (CI). The chi-squared test of Q and the I2 statistic were calculated to assess heterogeneity across the studies and to determine the appropriateness of applying either a random-effects model or fixed-effects model to the pooling process. Influence analyses were conducted to investigate the relative influence of each individual study on the pooled effect size using R package ‘meta’ metainf function. To measure publication bias, Egger’s and Begg’s tests and a funnel plot, for which significance was *p* < 0.05, were performed.

### TCGA, GTEx, and GEO gene expression data integration and differentially expressed genes (DEGs) identification

RNA-Seq data of 356 ovarian cancer and 180 normal tissue samples were downloaded from Genotype-Tissue Expression (GTEx) (https://www.gtexportal.org/home/) and TCGA via NCI’s Genomic Data Commons (GDC) portal (https://portal.gdc.cancer.gov/). Besides, five gene expression profiles - Datasets GSE137238, GSE101108, GSE101948, GSE143897, and GSE132107 - were downloaded from GEO, with a sample size of 323 ovarian cancer and 8 normal tissue samples. We integrated the data and ran ‘ComBat-seq’ in the R package ‘SVAseq’ (Version 3.38; http://bioconductor.org/packages/release/bioc/html/sva.html) to correct the batch effect. ‘DESeq2’ (Version 1.30. 1[24]; http://bioconductor.org/packages/release/bioc/html/DESeq2.html) was used to detect the differential gene expression between ovarian cancer and normal ovarian tissue samples.

### Proteomic analysis

Proteomic data were downloaded from The National Cancer Institute’s Clinical Proteomic Tumor Analysis Consortium (CPTAC) (https://proteomics.cancer.gov/programs/cptac) [25]. After quality evaluation, a total of 84 ovarian cancer tissue and 22 normal tissue samples from the CPTAC Ovarian Cancer Confirmatory Study were retained for further proteomic study. Differentially expressed proteins (DEPs) between normal and tumor tissues were identified by R package ‘Limma’ with a FDR < 0.05 cut-off criteria.

### Survival analysis

Overall survival was computed as the number of years between the year of diagnosis and the year of death from all causes, the date of the last follow-up, or 5-year censored survival data. Kaplan–Meier curves comparing overall survival according to subgroups divided by expression of *KAZN* and a log-rank test were used to assess the survival distributions across the subgroups. Samples were divided into either “high” or “low” groups with the cutoff at the lower quartile and upper quartile of *KAZN* gene expression. In log-rank, *p* < 0.05 was considered statistically significant. The survival curve was plotted by R package ‘survival’ and ‘survminer’.

### Cox regressions analysis and ROC curve

The relationship between *KAZN* mRNA and patients’ overall survival was analyzed by univariate Cox regression using R package ‘survival’ and ‘survminer’. The forest plot for the *KAZN* hazard ratios and confidence intervals was created by ggplot2. To assess the performance of the gene risk model and compare the prognostic value, the time-dependent receiver operating characteristic (ROC) curve for this model was plotted using R package ‘survivalROC’.

### Methylation level and mRNA expression level correlation analysis

The DNA methylation data (Illumina Human Methylation 27 k) of ovarian cancer (*n* = 601) and normal tissues (*n* = 12) and corresponding clinical information were obtained from TCGA (https://portal.gdc.cancer.gov/). The obtained DNA methylation data were further analyzed using Perl script and R package ‘ggpubr’. The Spearman rank correlation coefficient test was used to examine statistical significance in differences between DNA methylation and the expression of *KAZN*. R package ‘ggplot2’ was used to plot.

### Tissue preparation

For the quantitative real-time PCR (qPCR) analyses, 6 ovarian cancer tissues (HGSOC) and 8 normal ovarian tissues were obtained from ovarian cancer patients in operation from The Third Affiliated Hospital, Harbin Medical University (Harbin, China).

### RNA extraction and qRT-PCR analyses

RNA isolation of ovarian tissue samples were conducted through TRizol reagent (Invitrogen) according to the manufacturer’s instructions. Total RNAs were reversely transcribed into cDNAs and then used to perform qRT-PCR with Biosystems (ABI) 7500 platform. *Homo sapiens* β-actin (beta ACTB) was selected as the internal reference gene. The primer sequences were as follows: *KAZN* forward 5′-GGCAGATGAAGGAGATGTTGGCGAAGG-3′; *KAZN* reverse 5′-CTCTCCTTGCGGTGCTGCTCATAGTTG-3′; β-actin forward 5′-GGGAAATCGTGCGTGACATT-3′; β-actin reverse 5′-GGAACCGCTCATTGCCAAT-3′. The *KAZN* gene expression was determined by the subtracting their threshold cycle values (CT) to CT of β-actin gene.

### *KAZN* gene methylation pattern analysis

GEO datasets Illumina Infinium 450 K BeadChips were used to detect the methylation sites. R package ‘minfi’ was used to check the different CpG sites. Online wANNOVAR webserver was used to annotate the CpG sites [26]. R package ‘pheatmap’ was used to generate the figure.

## Results

### Assessment of *KAZN* mRNA level in ovarian cancer, based on gene expression omnibus (GEO) datasets

**Fig. 1:**
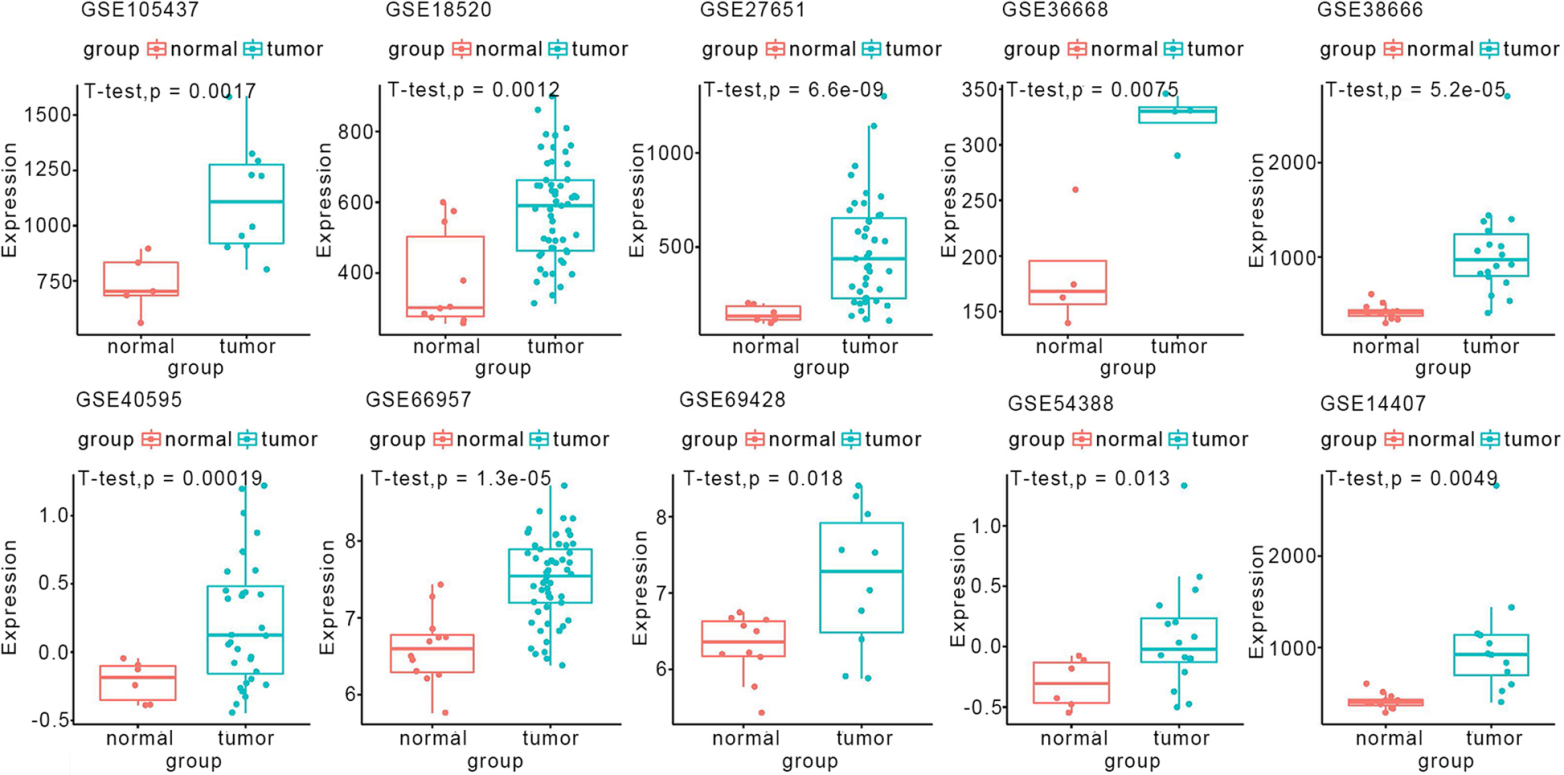
Expression of *KAZN* in ovarian cancer tissues and normal tissues based on Gene Expression Omnibus datasets. The expressions levels of *KAZN* are up-regulated in GEO datasets GSE105437, GSE18520, GSE27651, GSE36668, GSE38666, GSE40595, GSE66957, GSE69428, GSE54388, and GSE14407

The expression data of *KAZN* in ovarian cancer were obtained through the GEO database. A total of 11 microarrays from the GEO database met the entry criteria. The features of the selected GEO datasets are depicted in Table 1. Clinical features of samples in each dataset were listed in supplementary material Table S1. Expression of *KAZN* was significantly increased in ovarian cancer tissues in GSE105437, GSE18520, GSE27651, GSE36668, GSE38666, GSE40595, GSE66957, GSE69428, GSE54388, and GSE14407 (*p* = 0.0017, *p* = 0.0012, *p* < 0.0001, *p* = 0.0075, *p* < 0.0001, *p* < 0.0001, *p* < 0.0001, *p* = 0.0177, *p* = 0.0133, and *p* = 0.0049, respectively) (Fig. 1); no statistical difference was detected in GSE29450 (*p* = 0.0549, supplemental file: Fig. S1).

**Table 1:** Features of the enrolled Gene Expression Omnibus datasets

Accession	GPL	Year	Tumor			Normal			*p*-value	Source
			N	M	SD	N	M	SD		
GSE105437	GPL570	2017	10	1121.506	246.6307	5	734.7216	132.1731	0.001701071	tissue
GSE18520	GPL570	2009	53	577.697	142.3088	10	379.0934	138.5165	0.001188975	tissue
GSE27651	GPL570	2011	43	475.5898	279.5294	6	145.5886	45.25907	6.603179e-09	tissue
GSE36668	GPL570	2012	4	323.7239	23.08583	4	183.7234	52.44505	0.007529066	tissue
GSE38666	GPL570	2012	18	1057.761	506.7567	12	419.1524	82.5453	5.18647e-05	tissue
GSE40595	GPL570	2012	32	0.2308594	0.4661838	6	-0.2144503	0.1490786	0.000188017	tissue
GSE66957	GPL15048	2015	57	7.500341	0.5333843	12	6.60236	0.4617553	1.310033e-05	tissue
GSE69428	GPL570	2015	10	7.175477	0.9314753	10	6.292184	0.4273923	0.01771169	tissue
GSE29450	GPL571	2011	10	7.175477	0.7607612	10	7.612039	0.4370185	0.05491613	tissue
GSE54388	GPL570	2014	16	0.08109442	0.4558437	6	-0.3042137	0.2037893	0.01326	tissue
GSE14407	GPL570	2009	12	1037.8184	618.19663	12	411.1272	85.17666	0.00489	tissue

### Meta-analysis of GEO datasets

**Fig. 2:**
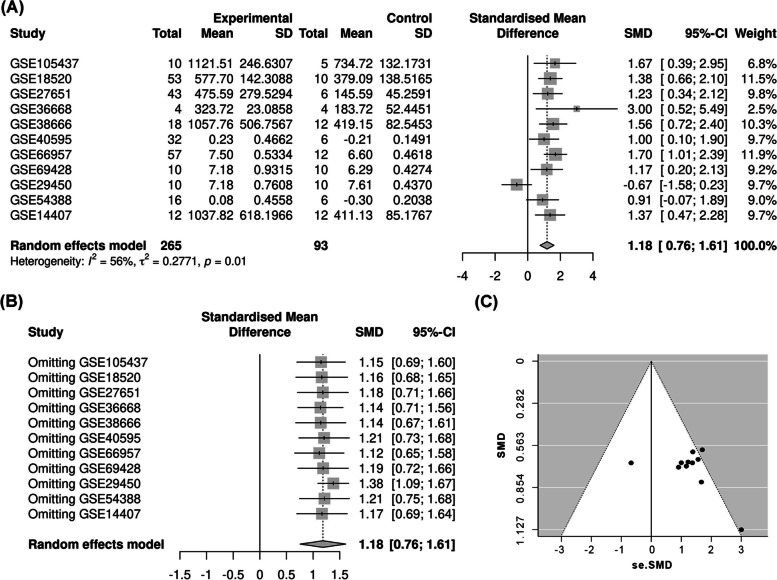
Meta-analysis of Gene Expression Omnibus (GEO) data. **A** Forest plot of GEO chips. The standard mean deviation is 0.99 (95% CI: 0.76, 1.61) with great heterogeneity (I^2^ = 56%, *p* = 0.01) showing that *KAZN* expression was markedly up-regulated in the ovarian cancer tissues. **B** Sensitivity analysis of GEO chips (*p* < 0.001). **C** A funnel plot for evaluating the publication bias of GEO chips (z = − 0.86, *p* = 0.3918)

Meta-analysis was conducted based on 11 included microarrays from the GEO database (Fig. 2A). Given the apparent heterogeneity (*p* = 0.01, I2 = 56%), a random-effects model was applied, and remarkable up-regulation (SMD = 1.18, 95% CI: 0.76, 1.61) of *KAZN* mRNA was found in ovarian cancer group.

Sensitivity analysis was performed to explore whether a particular microarray played a vital role in significant heterogeneity. By removing an individual study per time of meta-analysis to assess the influence of each study, the result showed that no study was found to have played a crucial role in any of the enrolled studies (overall effect *p* < 0.001, Fig. 2B). A funnel plot showed that no evidence of publication bias was observed for this analysis (*p* = 0.3918, Fig. 2C).

### Validation of *KAZN* expression by qRT-PCR

**Fig. 3:**
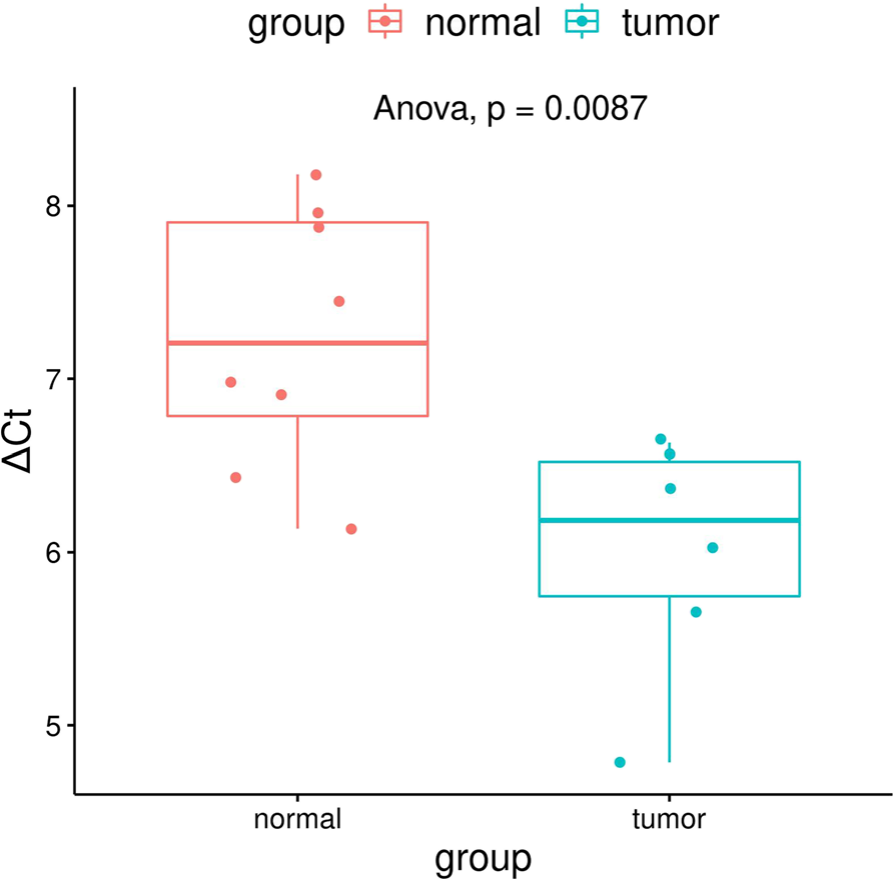
Quantitative real-time PCR analysis the expression of *KAZN* in human ovarian cancer tissues and normal ovarian tissues. ΔCt was used to determine the relative amounts of mRNA. There is Significant difference in 8 normal ovarian tissues and 6 tumor tissues by ANOVA statistical test, *p* = 0.0087

We used qRT-PCR to detect the expression levels of the *KAZN* in 14 human samples (8 normal ovarian tissues and 6 tumor tissues). The results showed that *KAZN* expression was significantly upregulated in ovarian cancer tissue compared with normal control (*p* = 0.0087, Fig. 3), which is. consistent with the results of GEO database.

### Up-regulation of *KAZN* affects overall survival in ovarian cancer

**Fig. 4:**
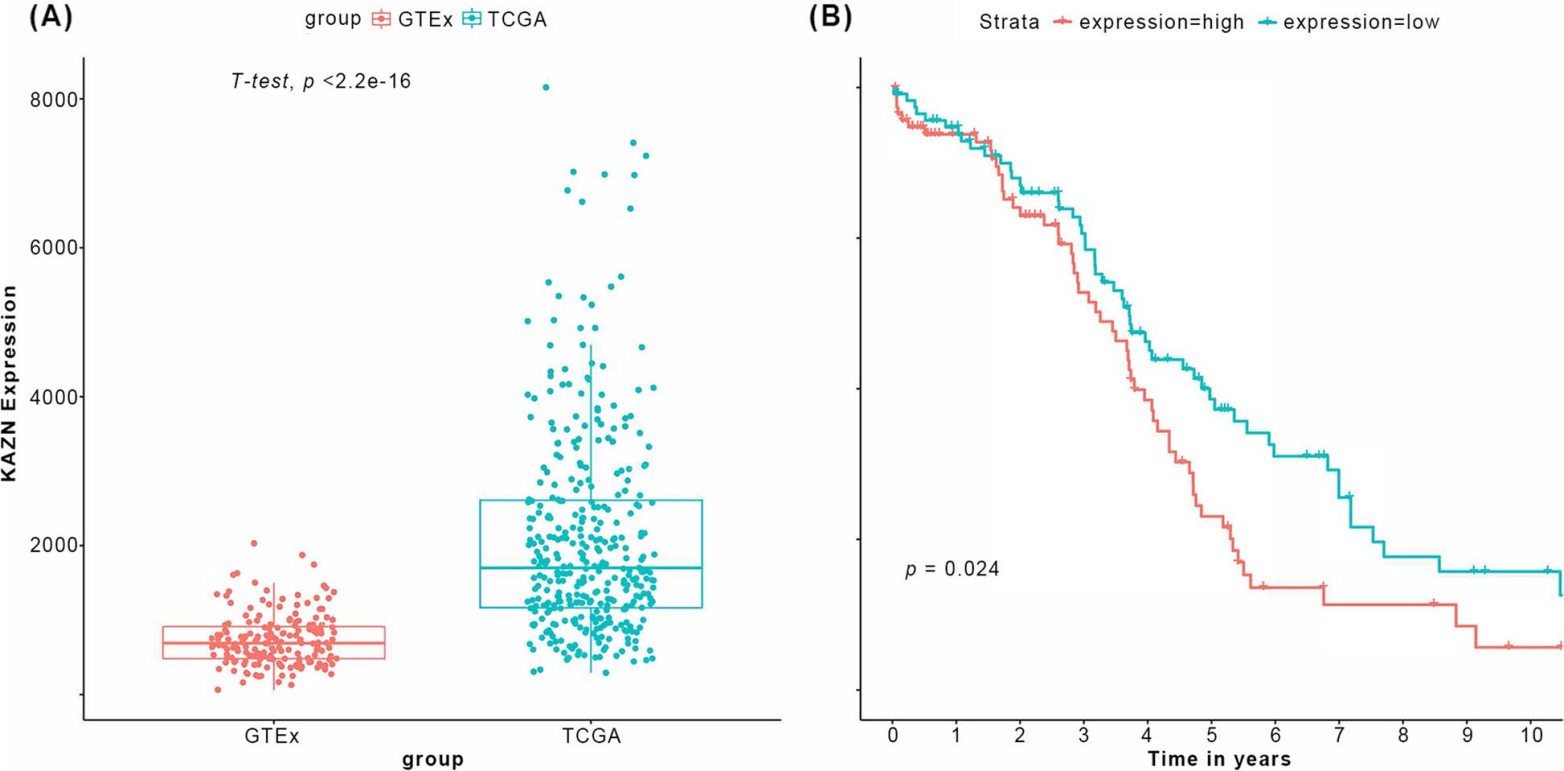
*KAZN* expression in ovarian cancer and survival analysis based on TCGA database. **A** Box plot, illustrating median expression levels of *KAZN* in normal ovarian tissue (Left, from GTEx) and ovarian tumors (Right, from TCGA). **B** Kaplan-Meier survival curve, illustrating overall survival for patients who had a tumor with up-regulated or down-regulated *KAZN*

TCGA ovarian cancer datasets have only cancer tissue samples, no normal tissue as a control, so we compared them to GTEx samples, which have expression data from normal ovary tissue of GTEx donors who did not have cancer. To eliminate the batch effect, we conducted ComBat-seq to integrate different sourced datasets [24]. The comprehensive analysis based on TCGA, GTEx, and GEO datasets showed that the *KAZN* mRNA expression was significantly differential, and the expression was up-regulated in the TCGA group compared to GTEx group (*p* < 0.0001, Fig. 4A).

To further study the clinical effects of *KAZN* in ovarian cancer, we divided the cases from TCGA OC datasets into four groups by the quantiles of *KAZN* counts, use quantile 1 and 4 as the *KAZN* high expression group and *KAZN* low expression group, and analyzed the survival status between the two groups. A Kaplan-Meier curve was used to identify the effects of the expression of *KAZN* on survival time and showed that the *KAZN* low expression group had significantly longer survival time than the *KAZN* high expression group (Fig. 4B). Univariate Cox regression analysis showed the association of *KAZN* mRNA with overall survival, demonstratig *KAZN* as a risk factor for OC (HR, 1.19, 95% CI, 1.03-1.37, *p* = 0.015, Fig. S2). Time-dependent ROC analysis indicated the prognostic accuracies were 0.652 at 6.8 years (Fig. S3).

### KAZN protein was up-regulated in ovarian cancer

**Fig. 5:**
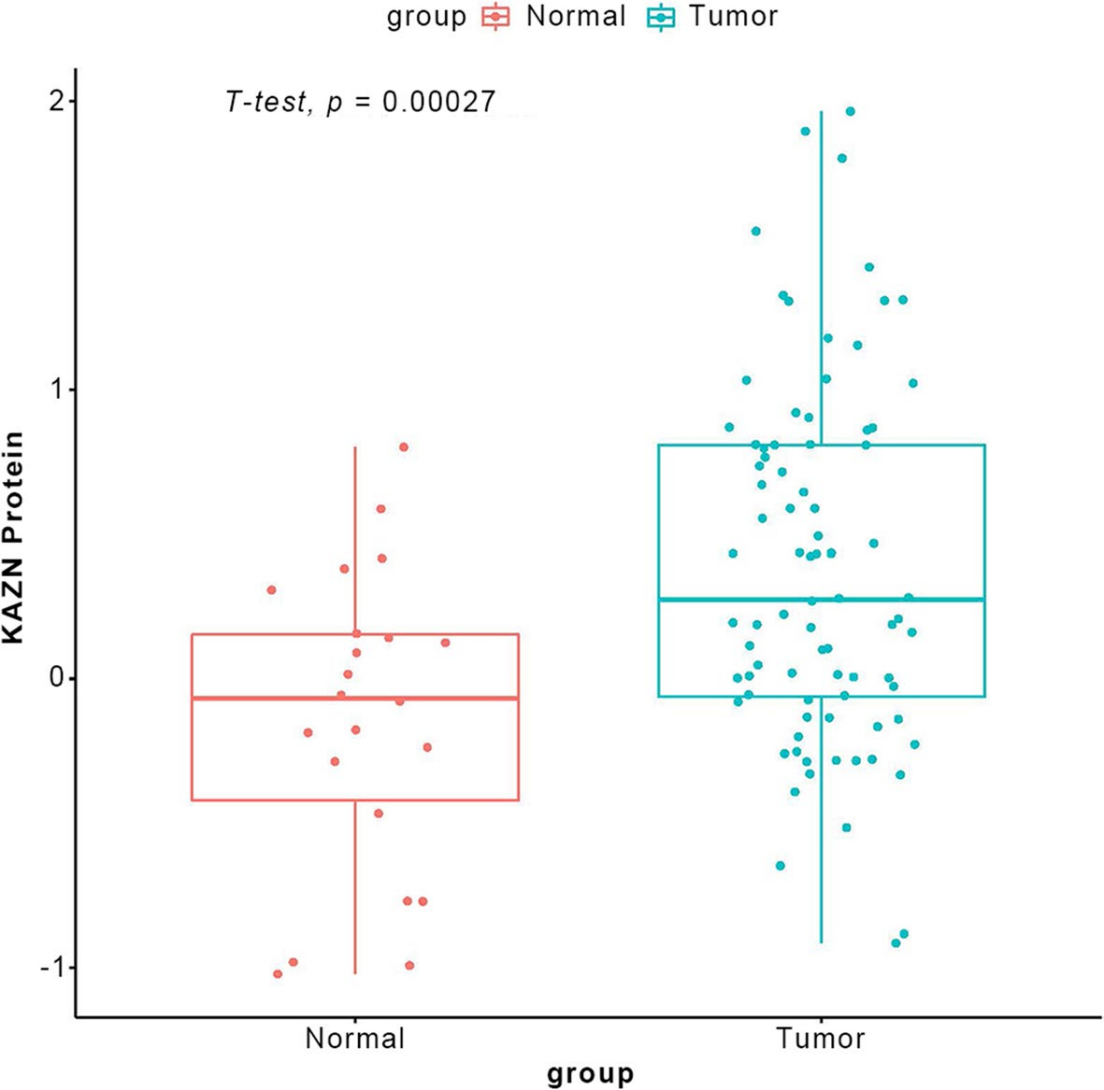
*KAZN* protein level in ovarian cancer. The box plot illustrates the median of the *KAZN* protein in normal ovarian tissue (Left) and ovarian tumors (Right) based on CPTAC datasets

The Clinical Proteomic Tumor Analysis Consortium (CPTAC) has produced huge amounts of cancer proteomics data providing unprecedented research opportunities. The dataset from CPTAC Ovarian Cancer Confirmatory Study contains 41 normal participants and 169 tumor participants, which was used to validate the *KAZN* protein expression in Ovarian cancer. The result showed that in the tumor group, the *KAZN* protein expression was significantly higher than in the normal group (*p* = 0.0027, Fig. 5). *KAZN* protein expression was consistent with the mRNA level in this study.

### Correlation of *KAZN* methylation level with mRNA expression in ovarian cancer

**Fig. 6:**
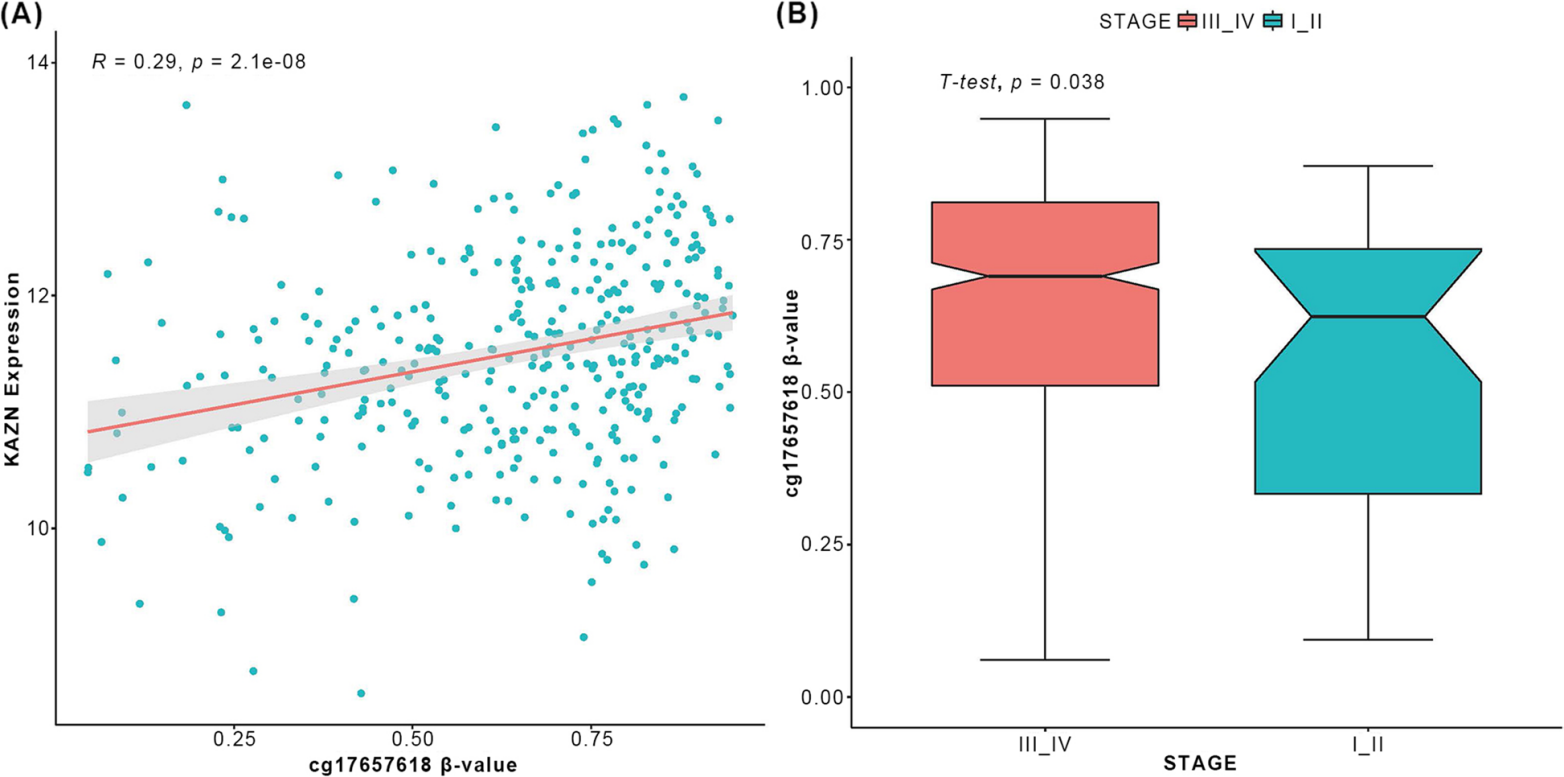
Correlation between *KAZN* methylation and mRNA expression and Correlation between *KAZN* methylation and clinical stage. **A** This plot illustrates that *KAZN* mRNA is positively correlated to cg17957618 methylation level (*R* = 0.29, *p* = 2.1e-08). **B** Boxplot for the cg17657618 methylation level between “stage I and II” group and “stage III and IV” group

Methylated CpG sites have a moderate to strong associations with gene expression changes across the phases in cancer-involved genes with specific functions. To study the correlation between the expression of *KAZN* and DNA methylation, we detected the DNA methylation level of CpG sites in the *KAZN* gene body region in TCGA datasets. From the TCGA database, we obtained 27 K DNA methylation array data of ovarian cancer, which contains 601 tumors and 12 normal samples. We detected the correlation between the expression of *KAZN* and cg17657618 and found the methylated CpG site cg17657618 positively correlated with the rising expression of the *KAZN* gene (Fig. 6A).

To detect the correlation between *KAZN* DNA methylation and the progress of OC, we divided all cases into two groups by stage of clinical traits. We compared the methylation level between the two groups. The result showed that the cg17657618 methylation level of the stage “I and II” group was significantly lower than the stage “III and IV” group (Fig. 6B). Survival analysis of cg17657618 showed a trend of longer survival time for the low CpG beta value group, though not statistically significant (*p* = 0.089) (Fig. S4), suggesting that *KAZN* methylation may have an important role in the development and outcome of ovarian cancer.

### The diagnostic value of *KAZN* methylation status in ovarian cancer

**Fig. 7:**
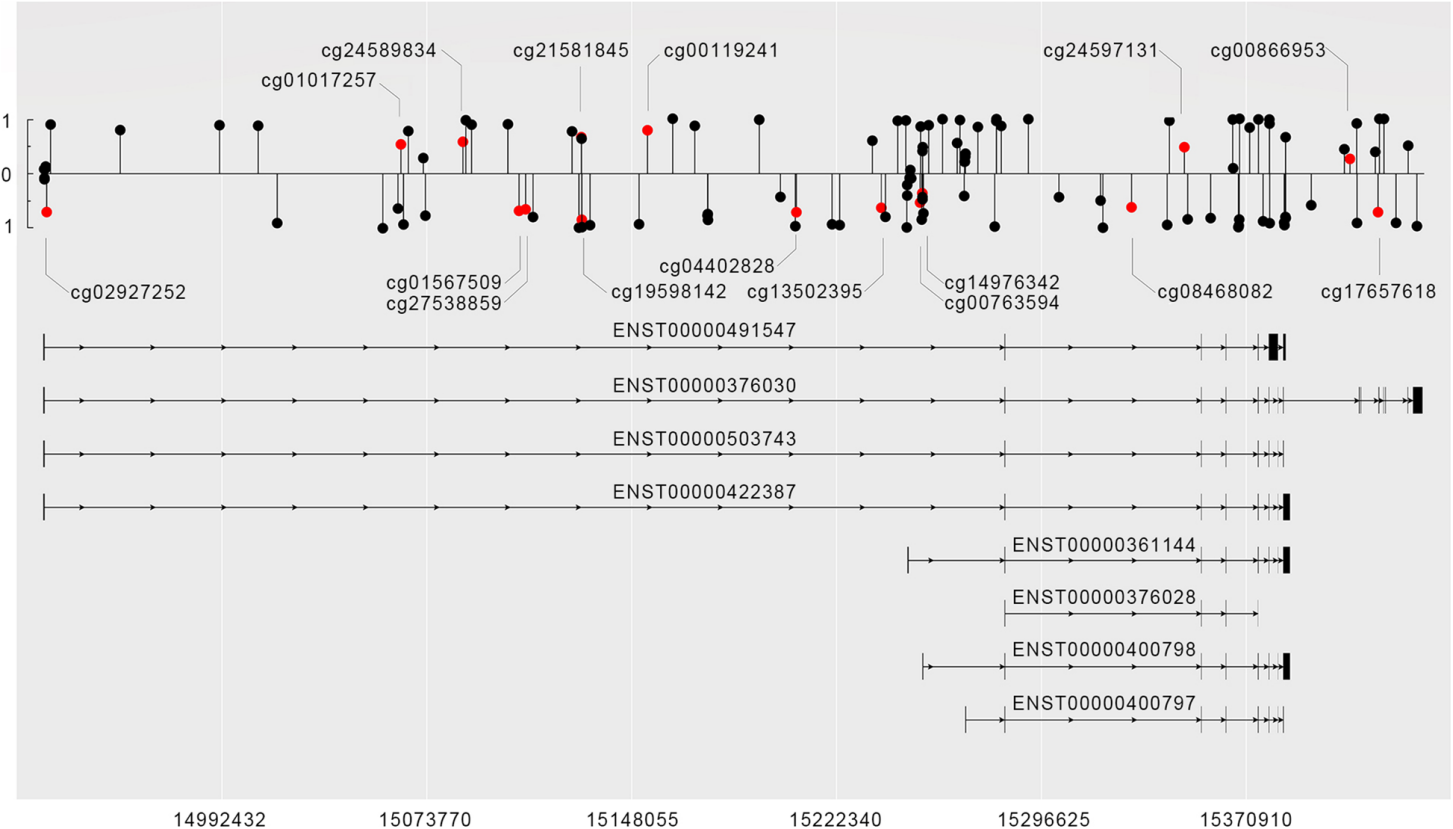
All CpG sites in *KAZN* gene body based on Illumina Infinium 450 K BeadChip. The upward ball-bar shape represents the CpG site in the forward strand. The downward ball-bar represents the CpG site in the reverse strand. The length of the bar represents the average methylation beta value. The red CpG sites are differentially methylated in the GSE146552 and GSE81224 datasets

**Fig. 8:**
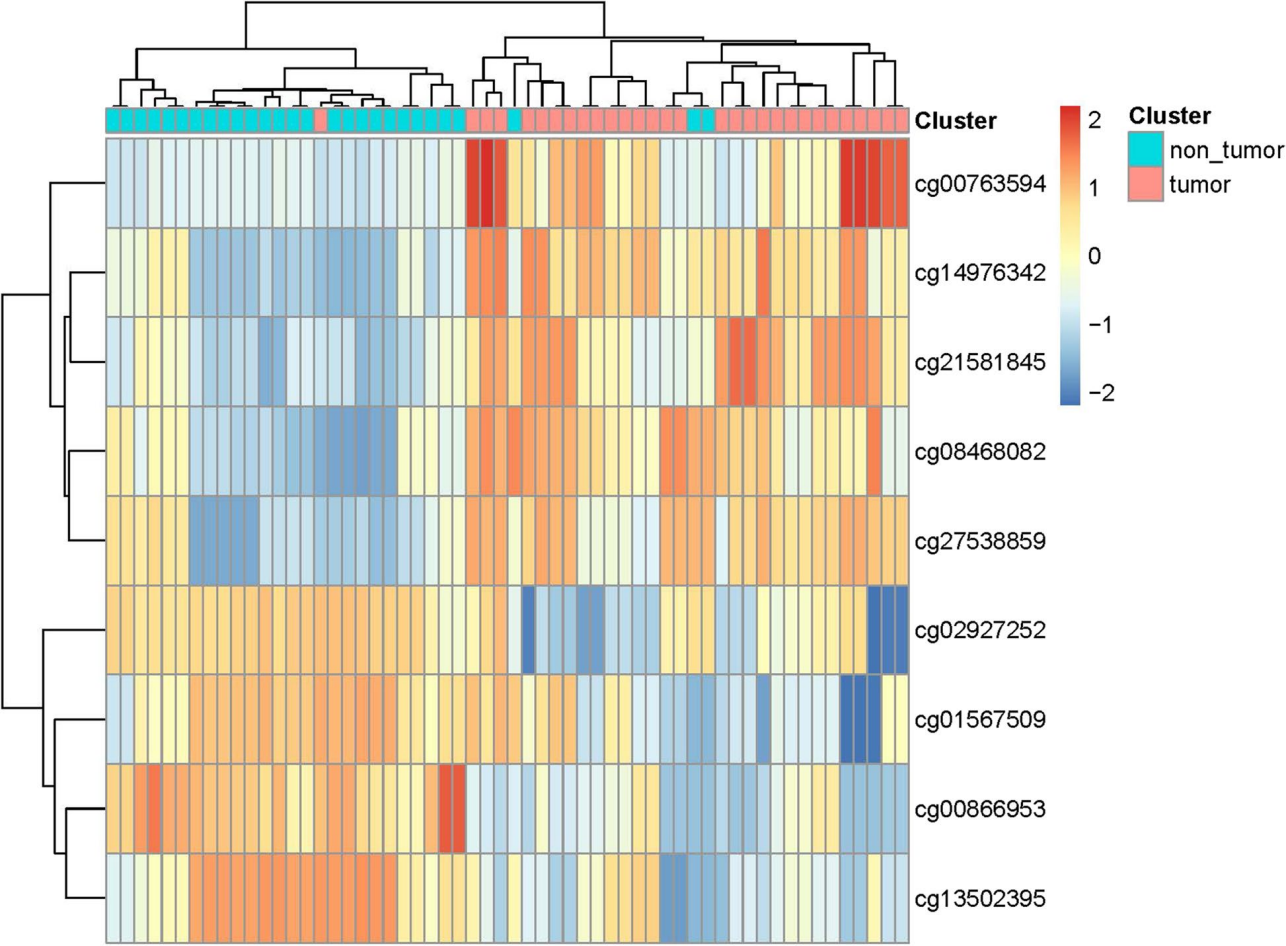
Pheatmap of 9 *KAZN* methylation CpG sites. The tumor (epithelial ovarian cancer) group and non-tumor (non-tumor, includes epithelial layer of normal ovary and fimbriae fallopian tubes) group have different methylation patterns

Illumina Infinium 450 K BeadChip covers 134 CpG sites for *KAZN*. The overview of all CpG sites for *KAZN* is shown in Fig. 7. In the GSE146552 and GSE81224 datasets, we found 13 and 12 differentially methylated CpG sites in the *KAZN* gene body region, respectively (details in supplemental Table S2). An unsupervised hierarchical clustering based on the 9 both different methylated CpG sites (cg00763594, cg00866953, cg01567509, cg02927252, cg08468082, cg13502395, cg14976342, cg21581845, cg27538859) in the two datasets in *KAZN* was constructed. The 9 CpG sites methylation pattern almost perfectly divided the samples of GSE146552 and GSE81224 into two groups, the tumor cluster, and the non-tumor cluster (Fig. 8), suggesting that the CpG pattern may potentially be used as a new biomarker for the diagnosis of ovarian cancer.

## Discussions

Previous studies have shown that *KAZN* is involved in multiple biological processes during development, such as cell proliferation and differentiation [17], as well as apoptosis [18], which prompted us to attempt identifying any possible associations of *KAZN* defects or abnormal expression levels with queer behaviors of cells, e.g., malignancies. To date, most of *KAZN* related studies have been focused on keratinocytes [15, 17, 27]. However, as *KAZN* is also expressed in many other tissues, its roles in health and diseases in general need to be investigated.

In the present study, we demonstrated that the expression of *KAZN* was significantly associated with OC. We compared 11 GEO microarray datasets to detect *KAZN* expression at the mRNA level and also conducted meta-analysis. *KAZN* mRNA was significantly up-regulated in OC in 10 GEO datasets, including 9 serous ovarian cancers and one unspecified ovarian cancer. In the remaining one dataset, which is clear cell cancer (supplemental file: Fig. S1), we did not find significant differences in *KAZN* expression, suggesting that the expression of *KAZN* may be correlated to the histological subtype of OC.

One of the merit of this work is the use of multiple data sources, which all indicated that *KAZN* was differentially expressed both on the mRNA level and on the protein level between ovarian cancer and normal tissues, with *KAZN* mRNA expression negatively correlated with survival time of the patients. *KAZN* can dually regulate proliferation and differentiation by Rho-dependent and –independent mechanisms [17], and also plays an important role in regulating cellular apoptosis by interacting with ARC and Bax [18]. During invasion and metastasis, cancer cells undergo changes in morphology and disruptions of cell connections. Overexpression of *KAZN* in keratinocytes can cause changes in cell shape and impair the assembly of intercellular junctions. As a component of the desmosome, *KAZN* also participates in the formation of cell connections [15, 17]. A recent study suggests that *KAZN* F is highly expressed in human cervical cancer tissues and could promote cell proliferation, migration and invasion in vitro by inhibiting apoptosis and facilitating epithelial-to-mesenchymal transition (EMT) [18]. It was further proved that *KAZN* F was directly regulated by miR-186, which influences the sensitivity of ovarian cancer cells to paclitaxel and cisplatin [28]. As such, it is reasonable to speculate that *KAZN* gene may participate in tumorigenesis, invasion, and metastasis of OC by affecting these key processes.

Growing evidence has shown that epigenetic changes are involved in cancer development and progression [22]. Indeed, DNA hypermethylation can lead to the silencing of tumor suppressor genes, whilst DNA hypomethylation can induce genomic instability, increasing transcription and facilitating protein activation [29]. A large number of putative suppressor genes that are silenced or activated by aberrant methylation have been identified in ovarian cancer, including hypermethylation of *OPCML*, *RASSF1*, *CDKN2B* as well as classical tumor suppressors *BRCA1*, *p16* and *MLH1*, and hypomethylation of *LINE-1, SLC6A12* and *PRAME* [30–38]*.* A recent study reported the association of CpG site cg17657618 with Endometriosis [39], but whether this CpG site may be associated with ovarian cancer was unknown. Our results based on TCGA human methylation 27 K BeadChips showed that cg17657618 was hypermethylated and positively correlated with the expression of the *KAZN* in OC. Analysis of the 450 K array dataset revealed 9 methylation sites that were differentially expressed in both datasets. The differentially expressed CpG sites, including 5 hypermethylated and 4 hypomethylated CpG sites in OC samples, may affect *KAZN* expression in different ways, whereby participating in the formation and progression of ovarian cancer. These findings all support the critical roles of *KAZN* gene expression and its methylation in OC occurrence and progress.

It is a common practice to use cluster analysis to divide patients into different groups based on the expression values of multiple genes or the top-ranked methylation CpG sites ß-value. A recent study discriminated EOC from normal ovarian tissues with high specificity and sensitivity based on the methylation of *RASSF1A*, *OPCML* and *HOXA9* [40]. In another study, methylation of *OPCML,* together with methylation of *RUNX3* and *TFPI2,* was demonstrated to be an early diagnostic marker for OC with higher sensitivity and specificity than classical CA125 [41]. However, our results based on single gene methylation CpG site ß-value were peculiar: the CpG methylation pattern of *KAZN* could be a new biomarker for predicting and diagnosing OC.

Previous studies have shown that *KAZN* has six splice variants, which are differentially expressed in a wide variety of cell types [15, 18]. As the transcripts are mostly tissue-specific and expressed in a temporal sequence, multiple methylation sites in the single gene are expected to play different roles at different stages in the development of ovarian cancer. In contrast to genetic changes, the relatively reversible character of the epigenetic alterations like DNA methylation determines that it has the potential to be artificially regulated. DNA methyltransferases (DNMTs) are responsible for the establishment and maintenance of the DNA methylation patterns on human genome. Hence, with continuous discovery of various global and specific DNMT inhibitors, CpG methylation sites of *KAZN* could be a new potential therapeutic target for ovarian cancer treatment. Further understanding of the function of the *KAZN* gene and the mechanistic relationship between *KAZN* expression and methylation in cancer will open a new horizon for the control of malignant diseases.

## Supplementary Information

**Additional file 1: Supplementary Table 1.** Clinical feature information of samples in each dataset.

**Additional file 2: Supplementary Table 2.** Differentially methylated CpG sites in the *KAZN* gene body region.

**Additional file 3: Supplementary Figure 1.** Expression of KAZN in ovarian cancer tissues and normal tissues based on Gene Expression Omnibus datasets. The expressions of KAZN are not significantly differential in GSE29450.

**Additional file 4: Supplementary Figure 2.** Forest plots of hazard ratios and their confidence intervals for the KAZN mRNA.

**Additional file 5: Supplementary Figure 3.** The time-dependent receiver operating characteristic (ROC) curve. The area under ROC curve (AUC) reached 0.652 at 6.8 year.

**Additional file 6: Supplementary Figure 4.** The Kaplan-Meier survival curve illustrate overall survival for patients who had tumor with hypermethylated or hypomethylated cg17657618.

## Data Availability

The dataset supporting the conclusions of this article is included within the article and its supplemental tables. Methylation and mRNA expression of ovarian cancer tissues were mined from The Cancer Genome Atlas (TCGA)(https://cancergenome.nih.gov/) and Gene expression omnibus (GEO, https://www.ncbi.nlm.nih.gov/geo/. The accession number is GSE105437, GSE18520, GSE27651, GSE36668, GSE38666, GSE40595, GSE66957, GSE69428, GSE54388, GSE14407, GSE137238, GSE101108, GSE101948, GSE143897, GSE132107, GSE146552 and GSE81224, respectively).
