# Supplementary figures and images for: KAZN as a diagnostic marker in ovarian cancer: a comprehensive analysis based on microarray, mRNA-sequencing, and methylation data
Source: BMC Cancer. 2022 Jun 16;22:662. doi: 10.1186/s12885-022-09747-2 (PMC9204993; doi:10.1186/s12885-022-09747-2)

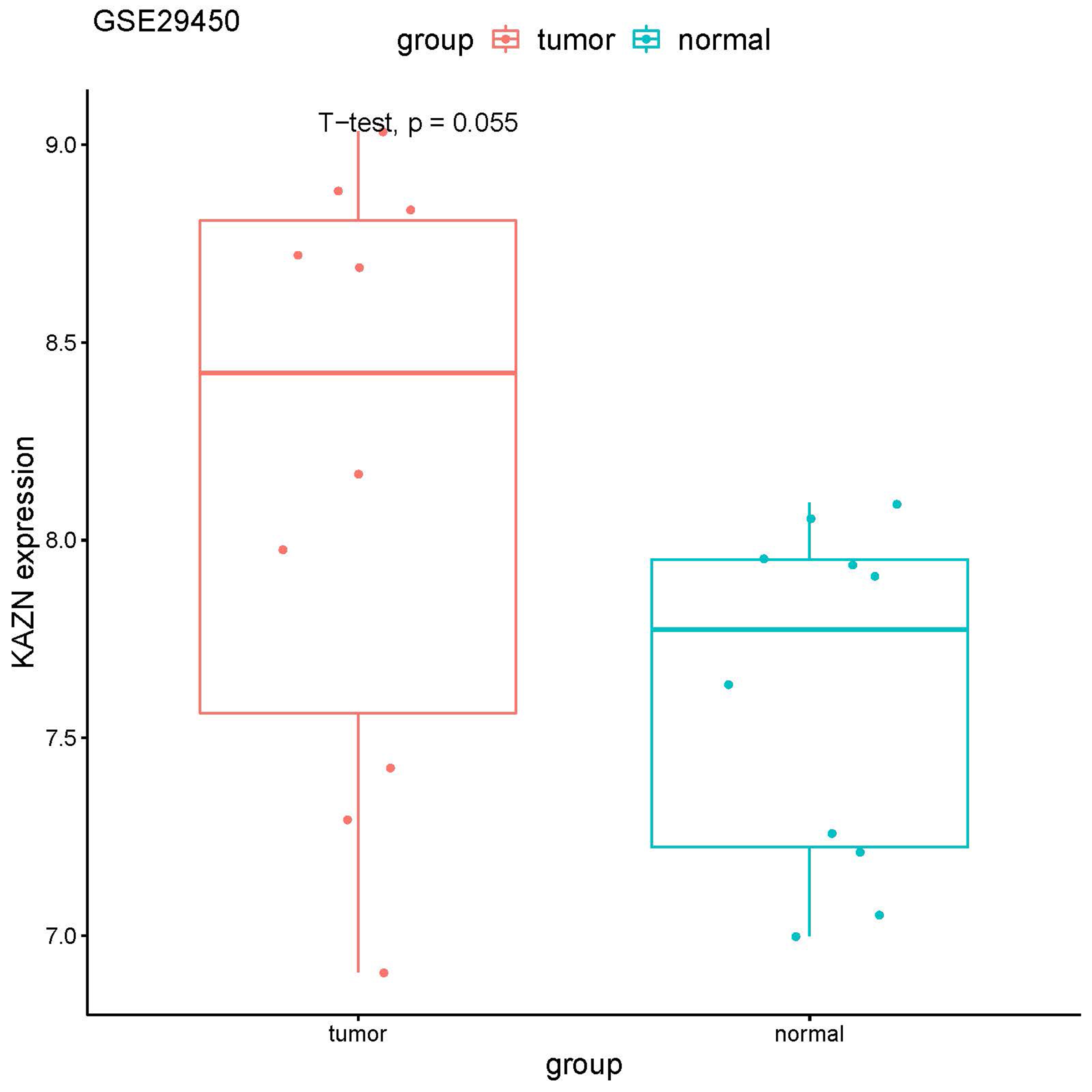

Supplement: Supplementary file 3 — Additional file 3: Supplementary Figure 1. Expression of KAZN in ovarian cancer tissues and normal tissues based on Gene Expression Omnibus datasets. The expressions of KAZN are not significantly differential in GSE29450. [file 12885_2022_9747_MOESM3_ESM.jpg]

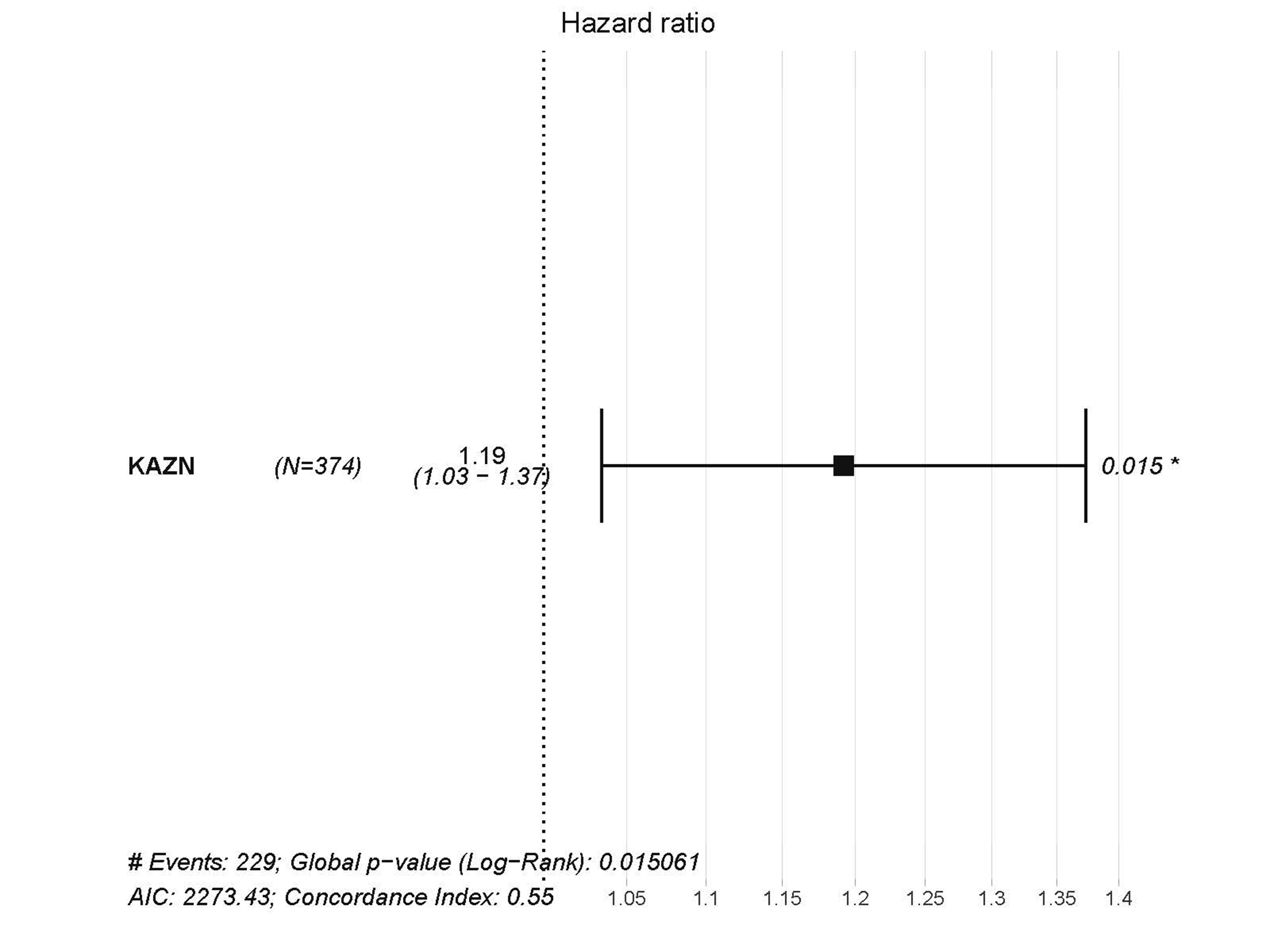

Supplement: Supplementary file 4 — Additional file 4: Supplementary Figure 2. Forest plots of hazard ratios and their confidence intervals for the KAZN mRNA. [file 12885_2022_9747_MOESM4_ESM.jpg]

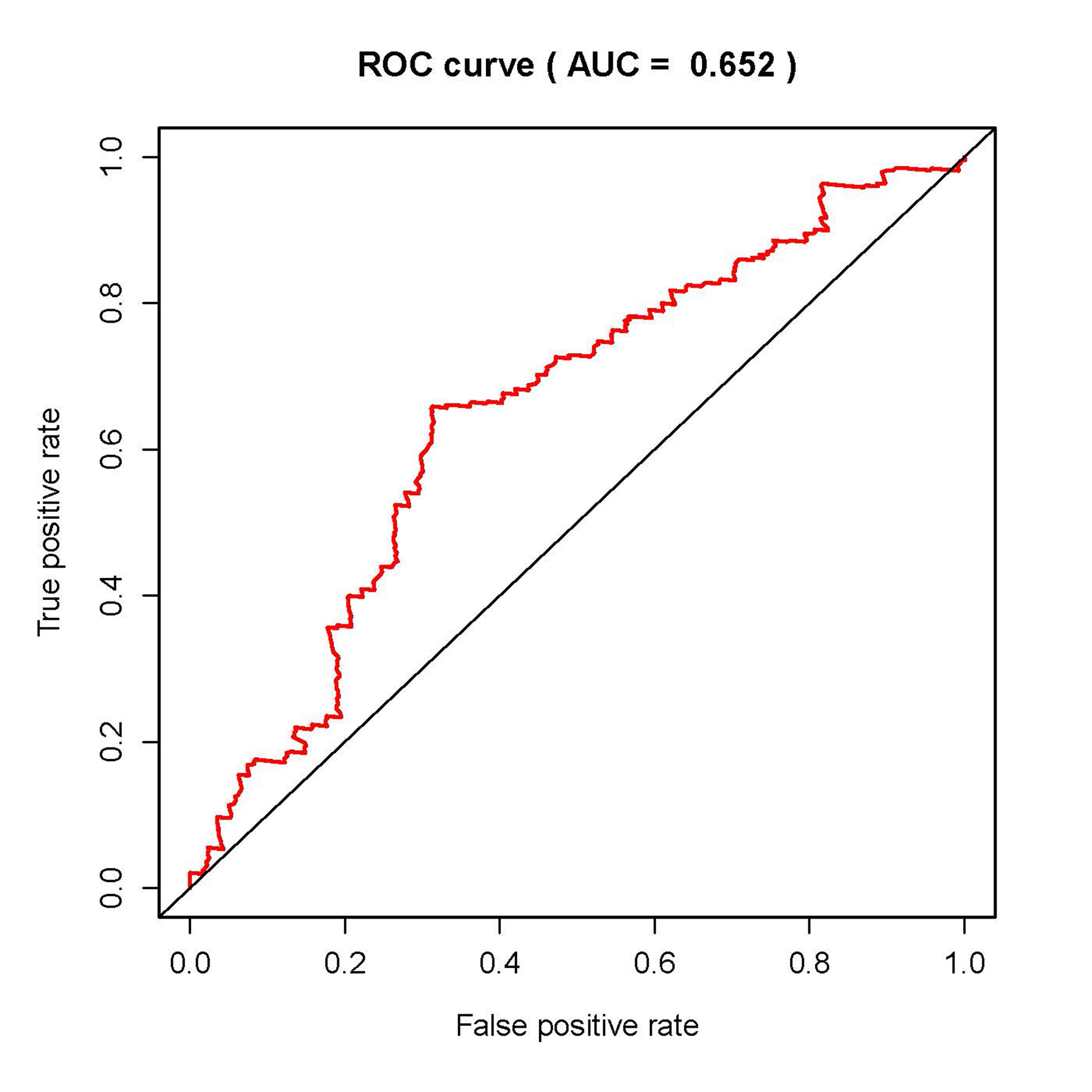

Supplement: Supplementary file 5 — Additional file 5: Supplementary Figure 3. The time-dependent receiver operating characteristic (ROC) curve. The area under ROC curve (AUC) reached 0.652 at 6.8 year. [file 12885_2022_9747_MOESM5_ESM.jpg]

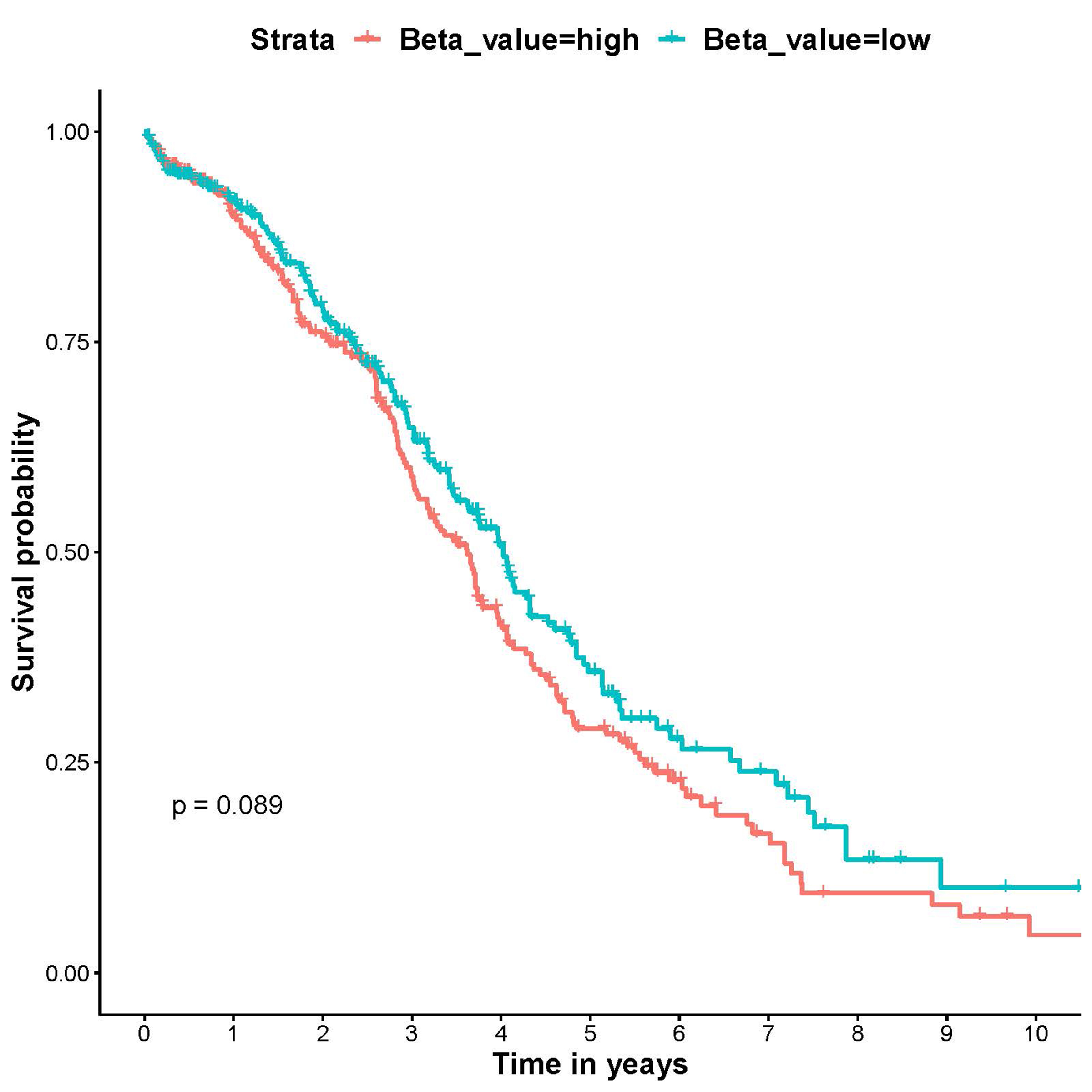

Supplement: Supplementary file 6 — Additional file 6: Supplementary Figure 4. The Kaplan-Meier survival curve illustrate overall survival for patients who had tumor with hypermethylated or hypomethylated cg17657618. [file 12885_2022_9747_MOESM6_ESM.jpg]
